# A Study on the Clinical Outcome of Abiraterone Acetate in Castration Resistant Prostate Cancer Patients

**Published:** 2018-01-01

**Authors:** Aloysius James, Bini Vincent, Akhila Sivadas, K. Pavithran

**Affiliations:** 1 Department of Pharmacy Practice, Amrita School of Pharmacy, Amrita Vishwa Vidyapeetham, Kochi, India; 2 Department of Medical Oncology and Hematology, Amrita Institute of Medical Sciences and Research Centre, Amrita Vishwa Vidyapeetham, Kochi, India

**Keywords:** Prostate-specific antigen (PSA), Abiraterone acetate (AA), Castration resistant prostate cancer (CRPCS)

## Abstract

**Background: **Abiraterone acetate was approved by FDA and EMA in April and September 2011, respectively for treatment of patients with casteration resistant prostate cancer and those previously treated with docetaxel. It is a selective inhibitor of androgen biosynthesis which potentially and irreversibly blocks CYP17, a crucial enzyme in oestrogen and testosterone synthesis.

**Materials and Methods: **This retrospective study was conducted to evaluate the safety and efficacy of abiraterone acetate in the treatment of castration resistant prostate cancer patients. Twenty-two male patients diagnosed with CRPC and experienced treatment failure with one or more lines of treatment (hormonal manipulation or chemotherapy) were selected and administered abiraterone acetate (1,000 mg daily) along with prednisone (5 mg twice daily).

**Results: **Out of 22 patients, 32% had a good response in reduction of PSA values, while 22% had progression in disease and 45% had a stable disease. Potassium, Haemoglobin, and serum sreatinine levels were not affected by the drug. Due to severe GI intolerance, the drug had to be stopped for one patient. The results of this study showed that abiraterone acetate significantly lowered the PSA values and prolonged progression- free survival in metastatic castration resistant prostate cancer patients who had progressed after first-line or second-line treatment. The overall average median survival and the median duration of drug exposure for CRPC who received AA was found to be 11.1 months [range 3−18]. Since AA plus prednisolone are available as oral dosage forms, they can be given in outpatient setting.

**Conclusion: **Abiraterone acetate is a drug of choice for CRPC and also for those who had previously received one or two chemotherapy regimens. Since it is a new therapeutic regimen, this study included small sample size, but there are a few studies indicating the therapeutic efficacy of AA among patients with castration-resistant prostate cancer.

## Introduction

 Prostate cancer is mostly found in male population in western countries, second to skin cancer^1^. The main treatment option is hormonal therapy which allows long lasting and effective control of advanced stage cancer symptoms. Hormonal therapy provides long lasting and effective control of cancer-related symptoms; however, in most patients with metastatic prostate cancer the disease will progress when it becomes resistant to androgen synthesis. The introduction of docetaxel for the treatment of CRPC turns a turning point resulting in increased response rate and biochemical control by decreasing the PSA (Prostate Specific Antigen) levels. Although the combination of docetaxel and prednisolone increases the survival time by decreasing the PSA value, there are only few other alternatives when progression occurs after the treatment with docetaxel^2^. Abiraterone acetate (AA), a selective inhibitor of androgen biosynthesis, is a prodrug of abiraterone that potently and irreversibly blocks CYP450C17. It is a crucial enzyme in testosterone and oestrogen synthesis, resulting in virtually undetectable serum and intratumoral androgens^3^. It works well along with prednisolone for metastatic castration resistant prostate cancer (mCRPC). AA plus prednisolone prolong overall survival rate in patients compared to other regimens^4^. The efficacy of treatment is measured using prostate specific antigen (PSA), a reliable sensitive and easy to measure biomarker. The most common adverse effects were associated with increased concentrations of mineralocorticoids resulting in hypokalemia, fluid retention, and hypertension^5^. Concomitant administration with corticosteroids reduces the incidence and severity of these reactions. This study attempted to evaluate the clinical outcome of abiraterone acetate in CRPC patients by measuring their PSA value and analyze the ADR profile of abiraterone acetate in lab parameters (Serum Creatinine, Potassium, CBC) in patients.

## MATERIALS AND METHODS

**Data collection**

This retrospective study was conducted in Medical Oncology Department in a Tertiary-care hospital. Data were reviewed from the electronic medical records and clinical details of those patients treated with AA between 2013 and 2015 were collected. Since it was a retrospective study, written informed consent was waived. 

**Patients and treatment**

Male patients with mCRPC who had disease progression after two or more regimens met the study inclusion criteria. Before administering AA, the patient was assessed for potassium level(less than 3.5mmol/L). AA was given as 4 tablets of 250mg per day one hour before or two hours after meal along with oral prednisolone 5mg twice a day. During the data collection period, 22 patients were treated with abiraterone acetate.

**Outcome measures**

The clinical and biological follow-up were scheduled every 15 days within the first three months of treatment and monthly afterwards until treatment discontinuation. All pertinent data including patients’ characteristics, disease progression at diagnosis, Gleason score, tumour classification, and sites of metastases were collected from medical records before chemotherapy and AA administration.

## Results

**Table 1:** Baseline characteristics

**Characteristics**		**Number**	**Median(range)**	
Age		60-88	70	
Initial Gleason Score				
5		2	9%	
7		6	27%	
8		10	45%	
9		4	18%	
Sites Of Metastasis before CT				
Bone only		17		77%
Multiple		2		10%
No Mets		3		14%
PSA before CT		0 - 3208	30.5	
PSA after progression from Chemotherapy		0.52 - 612	19.3	
PSA before AA		0.02 - 413	23.75	
PSA after progression from AA		10.781 - 864	40	
Lines of CT before AA		1	1	
		2	10	
		3	6	
		4	4	
Adverse Drug Reactions				
Serum Creatinine	Before	0.58 - 3.55	1.3	
	After	0.56 - 6.77	1.12	
Potassium	Before	3.4 - 5.1	4.1	
	After	3.5 - 5.9	4	
Haemoglobin	Before	2.35 - 11.3	11.5	
	After	2.34 - 12.9	10.5	

**Table 2:** Treatment duration of Abiraterone Acetate

**Months**	**No.**	**Percentage (%)**
≤3months	5	23
3-6 months	10	45
>6months	7	32

For the effective analysis, survival time was calculated in two different manners: from the beginning of chemotherapy, defined as the time interval, between the start of first-line chemotherapy and before starting AA. Meanwhile, the effect of AA was also evaluated and the date of death was recorded. 

## Discussion

 Since abiraterone acetate was only used for the treatment of castration -resistant prostate cancer, this study included small sample size. 

The introduction of docetaxel for the treatment of CRPC was a turning point since it resulted in an increased response rate by decreasing the PSA levels. Thus, the combination of docetaxel and prednisolone increased the survival time by decreasing the PSA value. There was no good alternative after progression from docetaxel^6^. In this study, we evaluated the effect of AA on castration- resistant prostate cancer and those patients who had progressed after one or two chemotherapy regimens. Frequent side effects related to the mineralocorticoid excess were potassium, haemoglobin, and serum creatinine levels. Abiraterone acetate was discontinued in only one patient due to an increase in creatinine level (6.6mg/dl), and no other major adverse drug reactions occurred. These results were in contrast to the results obtained in the study conducted by Joan Carles et al^7^. Initial Gleason score was found to 

be above 8 (63%) in most patients; these results were similar to the study done by N Masahiko et al^7^. 77% of patients had bone metastasis, and 3 patients had no metastasis. Out of 22 patients, 16 had 2 - 3 previous chemotherapy regimens before starting abiraterone. 

Median PSA value before chemotherapy was found to be 30.5 ng/ml [0 - 3208] and before abiraterone acetate was reported 23.75ng/ml. Median PSA value just before progression after starting abiraterone acetate was 19.3ng/ml [0.02 - 413]. This result shows that there is a significant decrease in the maximum value (3208 ng/ml to 413 ng/ml). Also, the overall survival rate after starting abiraterone acetate was significantly higher. 45 % of patients had 3 - 6 months of overall survival rate, 32 % had more than 6 months of survival rate without progression, and 23 % had progression after starting the drug. The overall average median survival and the median duration of drug exposure for CRPC who received AA was found to be 11.1 months [range 3−18]. Since AA plus prednisolone are available as oral dosage forms, they can be given in outpatient setting. 

## CONCLUSION

 Abiraterone acetate is a drug of choice for CRPC and also for those who had previously received one or two chemotherapy regimens. Since it is a new therapeutic regimen, this study included small sample size, but there are few studies indicating the therapeutic efficacy of AA among patients with castration-resistant and newly diagnosed patients with metastatic prostate cancer. 
